# Steady morphokinetic progression is an independent predictor of live birth: a descriptive reference for euploid embryos

**DOI:** 10.1093/hropen/hoae059

**Published:** 2024-10-10

**Authors:** Aşina Bayram, Ibrahim Elkhatib, Erkan Kalafat, Andrea Abdala, Virginia Ferracuti, Laura Melado, Barbara Lawrenz, Human Fatemi, Daniela Nogueira

**Affiliations:** IVF Department, ART Fertility Clinics, Abu Dhabi, UAE; Department of Reproductive Medicine, UZ Ghent, Ghent, Belgium; IVF Department, ART Fertility Clinics, Abu Dhabi, UAE; School of Biosciences, University of Kent, Canterbury, UK; IVF Department, ART Fertility Clinics, Abu Dhabi, UAE; Division of Reproductive Endocrinology and Infertility, Koc University School of Medicine, Istanbul, Turkey; IVF Department, ART Fertility Clinics, Abu Dhabi, UAE; IVF Department, ART Fertility Clinics, Abu Dhabi, UAE; IVF Department, ART Fertility Clinics, Abu Dhabi, UAE; IVF Department, ART Fertility Clinics, Abu Dhabi, UAE; Department of Reproductive Medicine, UZ Ghent, Ghent, Belgium; IVF Department, ART Fertility Clinics, Abu Dhabi, UAE; IVF Department, ART Fertility Clinics, Abu Dhabi, UAE; Inovie Fertilité, France

**Keywords:** time-lapse, morphokinetics, PGT-A, euploidy, live birth, pregnancy loss, embryo development

## Abstract

**STUDY QUESTION:**

Can modelling the longitudinal morphokinetic pattern of euploid embryos during time-lapse monitoring (TLM) be helpful for selecting embryos with the highest live birth potential?

**SUMMARY ANSWER:**

Longitudinal reference ranges of morphokinetic development of euploid embryos have been identified, and embryos with steadier progression during TLM are associated with higher chances of live birth.

**WHAT IS KNOWN ALREADY:**

TLM imaging is increasingly adopted by fertility clinics as an attempt to improve the ability of selecting embryos with the highest potential for implantation. Many markers of embryonic morphokinetics have been incorporated into decision algorithms for embryo (de)selection. However, longitudinal changes during this temporal process, and the impact of such changes on embryonic competence remain unknown. Aiming to model the reference ranges of morphokinetic development of euploid embryos and using it as a single longitudinal trajectory might provide an additive value to the blastocyst morphological grade in identifying highly competent embryos.

**STUDY DESIGN, SIZE, DURATION:**

This observational, retrospective cohort study was performed in a single IVF clinic between October 2017 and June 2021 and included only autologous single euploid frozen embryo transfers (seFET).

**PARTICIPANTS/MATERIALS, SETTING, METHODS:**

Reference ranges were developed from [hours post-insemination (hpi)] of the standard morphokinetic parameters of euploid embryos assessed as tPB2, tPNa, tPNf, t2–t9, tSC, tM, tSB, and tB. Variance in morphokinetic patterns was measured and reported as morphokinetic variance score (MVS). Nuclear errors (micronucleation, binucleation, and multinucleation) were annotated when present in at least one blastomere at the two- or four-cell stages. The blastocyst grade of expansion, trophectoderm (TE), and inner cell mass (ICM) were assessed immediately before biopsy using Gardner’s criteria. Pre-implantation genetic diagnosis for aneuploidy (PGT-A) was performed by next-generation sequencing. All euploid embryos were singly transferred in a frozen transferred cycle and outcomes were assessed as live birth, pregnancy loss, or not pregnant. Association of MVS with live birth was investigated with regression analyses.

**MAIN RESULTS AND THE ROLE OF CHANCE:**

TLM data from 340 seFET blastocysts were included in the study, of which 189 (55.6%) resulted in a live birth. The median time for euploid embryos to reach blastulation was 109.9 hpi (95% CI: 98.8–121.0 hpi). The MVS was calculated from the variance in time taken for the embryo to reach all morphokinetic points and reflects the total morphokinetic variability it exhibits during its development. Embryos with more erratic kinetics, i.e. higher morphokinetic variance, had higher rates of pregnancy loss (*P* = 0.004) and no pregnancy (*P* < 0.001) compared to embryos with steadier morphokinetic patterns. In the multivariable analysis adjusting for ICM, TE grade, presence of nuclear errors, and time of blastulation, MVS was independently associated with live birth (odds ratio [OR]: 0.62, 95% CI: 0.46–0.84, *P* = 0.002) along with ICM quality. Live birth rate of embryos with the same ICM grading but different morphokinetic variance patterns differed significantly. Live birth rates of embryos exhibiting low MVS with ICM grades A, B, and C were 85%, 76%, and 67%, respectively. However, ICM grades A, B, and C embryos with high MVS had live birth rates of 65%, 48%, and 21% (*P* < 0.001). The addition of the MVS to embryo morphology score (ICM and TE grading) significantly improved the model’s AUC value (0.67 vs 0.62, *P* = 0.015) and this finding persisted through repeat cross-validation (0.64 ± 0.08 vs 0.60 ± 0.07, *P* < 0.001).

**LIMITATIONS, REASONS FOR CAUTION:**

The exclusion of IVF cases limits, for now, the utility of the model to only ICSI-derived embryos. The utility of these reference ranges and the association of MVS with various clinical outcomes should be further investigated.

**WIDER IMPLICATIONS OF THE FINDINGS:**

We have developed reference ranges for morphokinetic development of euploid embryos and a marker for measuring total morphokinetic variability exhibited by developed blastocysts. Longitudinal assessment of embryonic morphokinetics rather than static time points may provide more insight about which embryos have higher live birth potential. The developed reference ranges and MVS show an association with live birth that is independent of known morphological factors and could emerge as a valuable tool in prioritizing embryos for transfer.

**STUDY FUNDING/COMPETING INTEREST(S):**

This study received no external funding. The authors declare no conflicting interests.

**TRIAL REGISTRATION NUMBER:**

N/A.

WHAT DOES THIS MEAN FOR PATIENTS? Effectively selecting the best embryo to transfer is crucial in IVF clinics to maximize the likelihood of a successful pregnancy. The current methods used to select embryos for transfer mainly rely on static observations under a microscope at fixed time intervals during embryonic development, which may overlook significant changes in morphology that occur in between measurements. Time-lapse monitoring systems offer continuous observation during embryo development, allowing for a more detailed understanding of dynamic changes in morphology (known as morphokinetics) that occur throughout this process.

The methodology in this study involved examining the success of single embryo transfers after time-lapse monitoring data had been collected during embryo development. These embryos were euploid, i.e. with the correct number of chromosomes as analysed by preimplantation genetic testing for aneuploidy (PGT-A). Using this data with the aim to improve embryo selection processes, we developed a novel ‘morphokinetic variance score’ that is based on measurements of the timing of various events that occur during normal embryo development. We found that embryos with steadier morphokinetic patterns during their development, as indicated by a low morphokinetic variance score, are more likely to result in successful pregnancies leading to live birth. The addition of the morphokinetic variance score to established markers already in use proposes an additional valuable metric for prioritizing embryos for transfer.

## Introduction

IVF clinics worldwide continue to select embryos for transfer predominantly based on the subjective assessment of morphological grading parameters, such as the number, size and symmetry of blastomeres, and the degree of fragmentation. These parameters rely on static observations at fixed time intervals during embryonic development (Alpha Scientists in Reproductive Medicine and ESHRE Special Interest Group of Embryology *et al.*, 2011). However, embryo status can change markedly within a few hours, and significant events may be missed between observational time points (Giménez *et al.*, 2023).

The inception of time-lapse monitoring (TLM) systems enables continuous monitoring during embryo development allowing the mapping of morphological changes without disruption of embryo culture (Ciray *et al.*, 2014). Since then, numerous morphological parameters such as multinucleation at cleavage stage, cleavage dysmorphisms, and blastocyst collapsing/re-expansion dynamics have been investigated and are thought to be predictive of sustained implantation and ploidy (Ergin *et al.*, 2014; Balakier *et al.*, 2016; Bodri *et al.*, 2016; Barrie *et al.*, 2017; Lagalla *et al.*, 2017; Desai *et al.*, 2018; ESHRE Working Group on Time-Lapse Technology *et al.*, 2020). Initial studies deploying TLM provided evidence suggesting that the kinetics of aneuploid embryos are delayed in comparison to euploid counterparts (Campbell *et al.*, 2013). Some studies have identified significant differences in morphokinetic parameters between euploid and aneuploid embryos (Campbell *et al.*, 2013; Desai *et al.*, 2018); however, the study outcomes were not reproducible with external datasets (Kramer *et al.*, 2014; Rienzi *et al.*, 2015; Zhang *et al.*, 2017), and no individual study could provide sufficient evidence to recommend the clinical use of TLM for embryo ploidy assessment (ESHRE Working Group on Time-Lapse Technology *et al.*, 2020; Bamford *et al.*, 2022). Rather, a trend for distinguishing aneuploid embryos using TLM has been provided once studies were gathered into a recent meta-analysis (Bamford *et al.*, 2022). In this meta-analysis, Bamford *et al.* (2022), suggested t8 (time to eight-cells) and t9 (time to nine-cells) as the parameters with minimal heterogeneity that could possibly be correlated to aneuploidy (Bamford *et al.*, 2022). These results support investigations suggesting that aneuploidy causes delayed embryo cytokinesis.

Embryos with aneuploid cells may exhibit altered morphological developmental patterns due to their aberrant genetic constitution carrying errors in nuclear-spindle migration and impacting cytokinesis (Magli *et al.*, 2007; Sfakianoudis *et al.*, 2021; McCoy *et al.*, 2023). However, the introduction of time-lapse revealed that euploid blastocysts may also exhibit irregular morphokinetics. The reasons for such events, although not yet completely understood, may be a result of a rescue mechanism by the exclusion of aneuploid cells by the embryo, or other gene expression/cellular mechanisms linked to cell adhesion and cellular communication (Lagalla *et al.*, 2017, 2020; Rienzi *et al.*, 2019; Ozbek *et al.*, 2021; De Martin *et al.*, 2024). Disturbed morphokinetics in euploid embryos could even be caused by stress linked to laboratory manipulations and conditions, which might be unperceived and unavoidable (Ramos‐Ibeas *et al.*, 2019; Swain, 2019). Moreover, it is important to recognize that a ‘typical’ morphokinetics of development for euploid blastocysts is imprecisely defined. Furthermore, average implantation rates after the transfer of a single euploid embryo do not exceed 60% in most settings and euploidy status does not preclude pregnancy loss. Therefore, an assumed normal chromosome constitution, although critical in predicting embryo implantation, is not the only characteristic determining embryo competence (Cimadomo *et al.*, 2023). Evaluating morphokinetic features could help in selecting, within the sibling euploid embryos, those that finally progress to live birth.

Indeed, to accomplish this, identified markers of embryonic kinetics have been incorporated into decision algorithms for embryo selection by using static imaging at specific time points (Barberet *et al.*, 2019; Khosravi *et al.*, 2019; VerMilyea *et al.*, 2020) or dynamic TLM imaging during embryonic development (Kan-Tor *et al.*, 2020; Duval *et al.*, 2023; Theilgaard Lassen *et al.*, 2023; Valera *et al.*, 2023). However, the longitudinal changes during this temporal process potentially influenced by intrinsic factors like the degree of mosaicism (Lee *et al.*, 2019; Martín *et al.*, 2021) or extrinsic factors such as culture conditions (Van Duijn *et al.*, 2022), their reference standards, and the impact of such changes on sustained implantation remain unknown. Our aim in this study was 2-fold. First, we aimed to establish a descriptive reference for morphokinetic development of euploid embryos. This reference serves a similar function as the reference standards of foetal biometric growth or postnatal growth charts. Such charts are used to define normal and abnormal foetal growth by assigning percentiles to static measurements. Second, we sought to evaluate the association between deviations from normal morphokinetics development and live birth outcomes after SET in frozen transfer cycles.

## Materials and methods

### Ethical approval

This project has the approval of the local ethics committee with the code REFA089 in 2023. The study was carried out between October 2017 and June 2021 in a tertiary referral fertility centre.

### Study design

This was an observational, retrospective cohort study, carried out in patients with primary or secondary infertility who underwent euploid single frozen embryo transfer (FET) cycles ‘freeze-all’ approach due to pre-implantation genetic diagnosis for aneuploidy (PGT-A). All embryos were assessed and morphokinetic parameters were annotated during TLM culture, together with nuclear errors at the two- and four-cell stages and morphological blastocyst grade of expansion, trophectoderm (TE), and inner cell mass (ICM). All euploid embryos were singly transferred in a frozen transferred cycle and pregnancy outcomes were assessed as live birth, pregnancy loss, or no pregnancy. Reference ranges were developed from [hours post-insemination (hpi)] of the standard assessed morphokinetic parameters (tPB2, tPNa, tPNf, t2–t9, tSC, tM, tSB, and tB). Variance in morphokinetic pattern was measured and reported as morphokinetic variance score (MVS). The association of MVS of euploid embryos with pregnancy outcomes was investigated with regression analyses.

### Ovarian stimulation and embryo culture conditions

Patients underwent ovarian stimulation using standard protocols with either recombinant FSH or HMG as described (La Marca and Sunkara, 2014). Oocytes were collected in Quinn’s Advantage Medium with HEPES, (SAGE, Målov, Denmark) supplemented with HSA (Vitrolife, Goteborg, Sweden) (HTF–HSA), and washed in Global Total LP medium for fertilization (CooperSurgical, Venlo, The Netherlands) after which they were cultured at 37°C, 6% CO_2_ and 5% O_2_ until denudation. After ICSI, oocytes were immediately cultured in Global Total LP medium (CooperSurgical, Venlo, The Netherlands) at 37°C, 6% CO_2_ and 5% O_2_ in the Embryoscope (ES) time-lapse incubator (Vitrolife, Goteborg, Sweden). After medium refreshment on Day 3, embryos were cultured until the blastocyst stage, and a TE biopsy was performed on Days 5–7 of the pre-implantation development.

### Morphokinetic time-lapse parameters

The time-lapse parameters from the time of second polar body extrusion to full blastocyst formation (tPB2–tB) were annotated in accordance with the recommendations of Ciray *et al.* (2014). The morphokinetic timings for all embryos started from the time of injection. Annotations were done by senior embryologists and meticulously reviewed by one senior experienced embryologist for the following time points. Pictures were taken every 20 min in embryoscope and the following were assessed: tPB2, time of the second polar body extrusion; tPNa, time at which both pronuclei were already visible; tPNf, time of pronuclear (PN) fading or the first frame where both PN can no longer be visualized; t2–t9: indicating the time to two to nine individual blastomeres; tSC, indicating the first frame in which any sign of compaction is present; tM, marking the end of the compaction process (the morula may be fully or partially compacted); tSB, time to the start of blastulation in which the cavity formation is initiated; and tB, time to the full blastocyst, indicating the last frame before the zona starts to thin. Nuclear errors such as micronucleation, binucleation, and multinucleation were annotated when present in at least one blastomere at two- and four-cell stages or annotated as ‘not present’ by the embryologist.

### TE biopsy and next-generation sequencing

Blastocyst evaluation before biopsy and the detailed protocols for TE biopsy and next-generation sequencing (NGS) were previously described (Abdala *et al.*, 2022). The embryo biopsy was performed on Days 5–7 post-insemination according to their rate of development. If the expansion of the blastocysts was not sufficient and/or the TE had few/scattered cells on the afternoon of D5, the biopsy was postponed to D6. The same applied from D6 to D7. Blastocysts were placed in a droplet containing HEPES medium (SAGE). The zona pellucida of expanded blastocysts was perforated by pulses of laser (OCTAX; NaviLase, Germany), keeping away from the ICM at the 6 or 12 o’clock position, and five to eight TE cells were taken from each blastocyst using the flicking method (Oliana *et al.*, 2017). TE cells were placed in 0.2-ml polymerase chain reaction tubes containing 2.5 µl phosphate-buffered saline (PBS). PBS was used for washing and handling of TE cells. To analyse biopsied TE samples, an NGS platform was used (ReproSeq; Thermo Fisher, Waltham, MA, USA) (Rubio *et al.*, 2019).

### Frozen embryo transfer (FET) cycle preparation

The choice of the endometrial preparation was at clinicians’ discretion and the patients’ preference, as no protocol has proven to be superior (Groenewoud *et al.*, 2013). For both endometrial preparation approaches (HRT–FET/natural cycle (NC)), baseline transvaginal ultrasound scans were performed on cycle day 2/3 to exclude uterine and ovarian pathology. Patients were monitored according to clinical standard protocol. No threshold of endometrial thickness was required. Embryo transfer was performed ∼120 h after either confirmed ovulation (in NC) or start of progesterone exposure (HRT).

### Blastocyst grading, vitrification, warming, and transfer procedure

The blastocyst grade of expansion, TE and ICM, were assessed immediately before biopsy and categorized using Gardner and Schoolcraft criteria (Gardner and Schoolcraft, 1999), being classified as A, B, or C for both ICM and TE, accordingly. Blastocyst vitrification and warming were performed using the Cryotop method (Kitazato, Shizuoka, Japan) (Kuwayama, 2007). Vitrification was done strictly 1 h after biopsy. A single euploid blastocyst was warmed and incubated for 2–4 h to allow blastocoele re-expansion prior to transfer. Embryo transfers were performed under abdominal ultrasound guidance as described previously (Bayram *et al.*, 2021).

### Statistical analysis

Variables will be presented as median and interquartile range (IQR) or number and percentage of total, regardless of distribution characteristics. Time to reach morphokinetic milestones was treated as a continuous dependent variable. Dependent variables were modelled as a function of morphology developmental stages and linear mixed-effect regression with natural cubic splines were employed. Candidate models were compared with likelihood ratio tests. Model intercepts were allowed to vary between different embryos and slopes were allowed to vary for the measurements of the same embryo. Mean curve and standard deviation curve were estimated separately using the approach suggested by Royston and Wright (2000). All models were checked for outliers, influential data points, normality of residuals, and absence of autocorrelation in residuals. Outliers were either kept or removed from the model after double-checking the veracity of the measurements. Model fit was assessed as described by Royston and Wright by checking the normality of estimated *Z*-scores across the fitted range and independency from morphokinetic key parameters. After creation of reference standards, *Z*-scores of each time point and variance of *Z*-scores for each embryo were calculated (Royston and Wright, 1998). Natural log of variance is presented as morphokinetics variance score and its influence on live birth rates was assessed with logistic regression analyses and receiver operating characteristics curves. AUC metric was subject to repeated 5-fold cross-validation over 100 iterations to obtain optimism adjusted AUC values. Created reference ranges are presented for most commonly used percentile points for each morphokinetic time point and an online calculator is created for easy calculation of percentiles and scores. The concept of MVS and case examples of low and high variance scores are shown in Supplementary File S1. All analyses were conducted using R for Statistical Computing software (Vienna, Austria).

## Results

Morphokinetic data from 340 euploid single embryo transfers (n = 327 patients) were included in the study. Of these, 189 (55.6%) resulted in a live birth, 48 (14.1%) resulted in pregnancy loss after a positive HCG test and 103 (30.3%) did not result in pregnancy. Baseline characteristics of the women enrolled are shown in Table 1. There were no significant differences among the groups in women’s age, anti-Müllerian hormone, endometrial thickness, infertility type, and duration (Table 1). Patients who achieved a live birth were leaner (Body mass index (BMI) IQR: 25.8 vs 28.1 and 27.8 of pregnancy loss and non-pregnant groups, respectively; *P* = 0.008) and were more likely to have an NC endometrial preparation approach (43.4% vs 20.8% and 35.9% of pregnancy loss and no pregnant groups, respectively; *P* = 0.014).

**Table 1.** Baseline patient and embryo characteristics stratified according to transfer outcomes.

Variables	Live birth (n = 189)	Pregnancy loss (n = 48)	No pregnancy (n = 103)	*P* value
**Age, years**	34.0 (30.0 to 38.0)	32.0 (28.0 to 35.5)	33.0 (29.0 to 37.0)	0.095
**BMI, kg/m^2^**	25.8 (22.6 to 30.1)	28.1 (24.5 to 33.0)	27.8 (24.3 to 32.2)	0.008
**AMH, ng/ml**	2.5 (1.8 to 3.9)	3.6 (1.9 to 5.2)	2.4 (1.5 to 3.6)	0.053
**Cycle type**				
HRT	107 (56.6)	38 (79.2)	66 (64.1)	0.014
Natural	82 (43.4)	10 (20.8)	37 (35.9)	
**Endometrial thickness, mm**	7.7 (7.1 to 8.7)	7.6 (6.7 to 8.5)	7.7 (6.8 to 8.4)	0.723
**Infertility type**				
Primary	95 (50.3)	27 (56.2)	47 (45.6)	0.525
Secondary	94 (49.7)	21 (43.8)	56 (54.4)	
**Infertility duration**	2.0 (1.0 to 4.0)	2.0 (1.0 to 4.2)	2.0 (1.0 to 4.0)	0.900
**ICM quality**				
A	45 (23.8)	5 (10.4)	13 (12.6)	<0.001
B	135 (71.4)	41 (85.4)	65 (63.1)	
C	9 (4.8)	2 (4.2)	25 (24.3)	
**TE quality**				
A	42 (22.2)	13 (27.1)	16 (15.5)	0.028
B	125 (66.1)	28 (58.3)	61 (59.2)	
C	22 (11.6)	7 (14.6)	26 (25.2)	
Nuclear error(s)* at 2-cell stage				
No	78 (41.3)	20 (41.7)	46 (44.7)	0.850
Yes	111 (58.7)	28 (58.3)	57 (55.3)	
Nuclear error(s)* at 4-cell stage				
No	175 (92.6)	39 (81.2)	88 (85.4)	0.036
Yes	14 (7.4)	9 (18.8)	15 (14.6)	

BMI: body mass index; AMH: anti-Müllerian hormone; ICM: inner cell mass; TE: trophectoderm.

Continuous variables are presented as median and interquartile range, count variables are presented as number and percentage of total. Comparisons are made with either Kruskal–Wallis test or chi-squared test. *Nuclear errors accounted for are micronucleation, binucleation, and multinucleation.

### Embryo quality characteristics related to live birth

The ICM quality assessed before TE-biopsy of the transferred blastocysts resulting in live birth was more often Grade A (23.8%) compared to the pregnancy loss (10.4%) and no pregnancy groups (12.6%) (*P* < 0.001) (Table 1). Lower TE quality at the time of biopsy had a significant negative association with live birth (22.2% vs 27.1% and 15.5%, *P* = 0.028). The embryos leading to live births were also less likely to show nuclear errors at the four-cell stage (7.4%) compared to the pregnancy loss (18.8%) and no pregnancy groups (14.6%), respectively (*P* = 0.036). There were no significant differences among the transferred euploid embryos in terms of the presence of nuclear errors at the two-cell stage (Table 1, *P* > 0.05).

### Reference curves of euploid embryo growth and MVS

**Figure 1.**
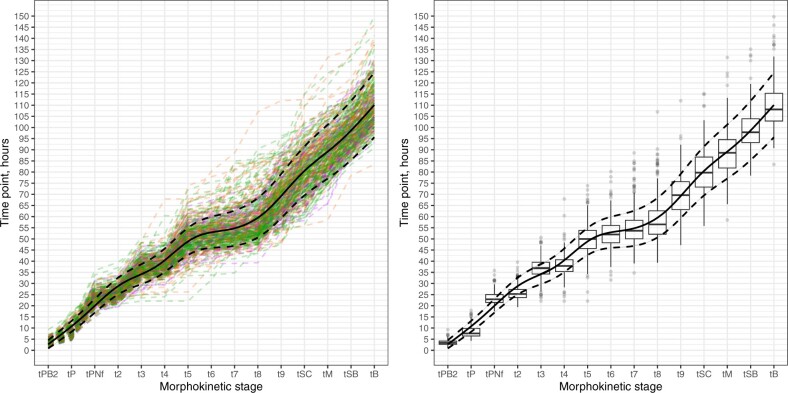
**Longitudinal trajectories of euploid embryo development**. Fitted regression model (solid line: mean; dashed lines: prediction intervals) with longitudinal trajectories of individual embryos (left) and boxplots of time values at each morphokinetic point (right). Longitudinal trajectories are colour-coded according to outcome (green: live birth; purple: pregnancy loss; orange: no pregnancy). tPB2, time of the second polar body extrusion; tPNa, time at which both pronuclei were already visible; tPNf, time of pronuclear (PN) fading or the first frame where both PN can no longer be visualized; t2–t9: time to two to nine individual blastomeres; tSC, first frame in which any sign of compaction is present; tM, end of the compaction process: the morula may be fully or partially compacted; tSB, start of blastulation in which the cavity formation is initiated; tB, full blastocyst: indicates the last frame before the zona starts to thin.

A natural cubic spline function with knots at each milestone provided the best fit to the data (Fig. 1). Reference curves were constructed, and the following percentiles are provided for each morphokinetic parameter, 3rd—5th—10th—25th—50th—75th—90th—95th—97th (Table 2). Estimated ‘normal’ ranges for morula development in euploid embryos (3rd to 97th percentile) were 79.6–98.1 h. This indicates the normal range for reaching the morula stage is on the 4th day and embryos failing to reach morula stage by 98 h.p.i may be delayed. Normal time point ranges for blastocyst development were 98.8–121.0 h with a median time to reach blastulation of 109.9 h, respectively. This indicates the normal range for reaching the blastulation stage is on the 5th day and embryos failing to reach blastulation by 121 h.p.i may be delayed. After construction of reference ranges, *Z*-scores of each developmental stage and the variance of *Z*-scores for each embryo were calculated. MVS was calculated as natural log of variance.

**Table 2.** Morphokinetic milestones reference ranges in hours for euploid embryos (3rd–97th percentiles).

Morphokinetic milestones*	3rd	5th	10th	25th	Median	75th	90th	95th	97th
tPB2	2.07	2.25	2.52	2.98	3.48	3.99	4.45	4.72	4.90
tPNa	6.44	6.67	7.03	7.63	8.30	8.97	9.57	9.93	10.16
tPNf	20.99	21.29	21.74	22.50	23.34	24.18	24.94	25.39	25.68
t2	22.92	23.28	23.84	24.76	25.79	26.82	27.75	28.30	28.66
t3	33.46	33.89	34.55	35.66	36.89	38.12	39.23	39.89	40.32
t4	34.31	34.81	35.59	36.89	38.33	39.78	41.08	41.86	42.36
t5	45.28	45.87	46.77	48.27	49.94	51.62	53.12	54.02	54.61
t6	47.14	47.80	48.84	50.56	52.47	54.38	56.11	57.14	57.81
t7	49.18	49.93	51.10	53.05	55.22	57.39	59.34	60.51	61.27
t8	51.94	52.79	54.11	56.30	58.74	61.17	63.37	64.68	65.53
t9	62.47	63.42	64.89	67.34	70.06	72.78	75.22	76.69	77.64
tSC	71.79	72.85	74.47	77.18	80.20	83.21	85.93	87.55	88.61
tM	79.66	80.82	82.61	85.60	88.92	92.25	95.24	97.03	98.19
tSB	88.85	90.13	92.09	95.37	99.02	102.67	105.95	107.91	109.19
tB	98.81	100.21	102.35	105.94	109.92	113.90	117.49	119.63	121.03

* Morphokinetic milestones: tPB2, time of the second polar body extrusion; tPNa, time at which both pronuclei were already visible; tPNf, time of pronuclear (PN) fading or the first frame where both PN can no longer be visualized; t2–t9: time to two to nine individual blastomeres; tSC, first frame in which any sign of compaction is present; tM, end of the compaction process, the morula may be fully or partially compacted; tSB, start of blastulation in which the cavity formation is initiated; tB, full blastocyst: indicates the last frame before the zona starts to thin.

Univariable regression analyses showed that lower ICM quality grade, lower TE quality grade, and presence of nuclear errors at four-cell stage decreased the odds of live birth (Table 3). The MVS was also significantly associated with the odds of live birth (OR: 0.59, 95% CI: 0.45–0.77, *P* < 0.001) (Table 3). In the multivariable analysis, only MVS and ICM quality had a significant effect on live birth. One standard deviation increase in MVS (∼0.9 points) was associated with 38% lower odds of live birth (OR: 0.62, 95% CI: 0.46–0.84; *P* = 0.002). In addition, embryos with ICM grades B and C had 49% and 79% lower odds of live birth, respectively (*P* = 0.049 and 0.009).

**Table 3.** Regression analysis of embryo characteristics associated with live birth.

Variables	OR (95% CI, *P*)	aOR (95% CI, *P*)**
**Expansion grade**		
BL3	Reference	Reference
BL4	1.61 (0.85–3.06, *P* = 0.145)	1.06 (0.52–2.17, *P* = 0.865)
BL5	1.64 (0.89–3.06, *P* = 0.115)	1.03 (0.51–2.07, *P* = 0.929)
**TE quality grade**		
A	Reference	Reference
B	0.97 (0.56–1.67, *P* = 0.912)	1.25 (0.68–2.31, *P* = 0.470)
C	0.46 (0.22–0.94, *P* = 0.034)	1.30 (0.51–3.33, *P* = 0.583)
**ICM quality grade**		
A	Reference	Reference
B	0.51 (0.27–0.92, *P* = 0.028)	0.51 (0.26–0.98, *P* = 0.049)
C	0.13 (0.05–0.33, *P* < 0.001)	0.21 (0.06–0.65, *P* = 0.009)
**Day of blastulation**		
Day 5	Reference	Reference
Day 6	1.03 (0.55–1.95, *P* = 0.918)	1.66 (0.82–3.42, *P* = 0.161)
Day 7	0.80 (0.03–20.24, *P* = 0.872)	1.86 (0.07–50.24, *P* = 0.673)
*Nuclear error(s) at 2-cell		
No	Reference	Reference
Yes	1.10 (0.72–1.70, *P* = 0.651)	1.19 (0.74–1.92, *P* = 0.468)
*Nuclear error(s) at 4-cell		
No	Reference	Reference
Yes	0.42 (0.21–0.84, *P* = 0.016)	0.48 (0.22–1.03, *P* = 0.064)
**MVS**	0.59 (0.45–0.77, *P* < 0.001)	0.62 (0.46–0.84, *P* = 0.002)

OR: odds ratio; aOR: adjusted odds ratio; CI: confidence interval; BL: blastocyst; ICM: inner-cell mass; TE: trophectoderm; MVS: morphokinetic variance score. Analyses are made with logistic regression.

* Nuclear errors accounted for are micronucleation, binucleation, and multinucleation.

** Multivariable model includes expansion grade, trophectoderm grade, inner-cell mass grade, day of blastulation, multinucleation, and morphokinetic variance score.

BL, blastocyst.

**Figure 2.**
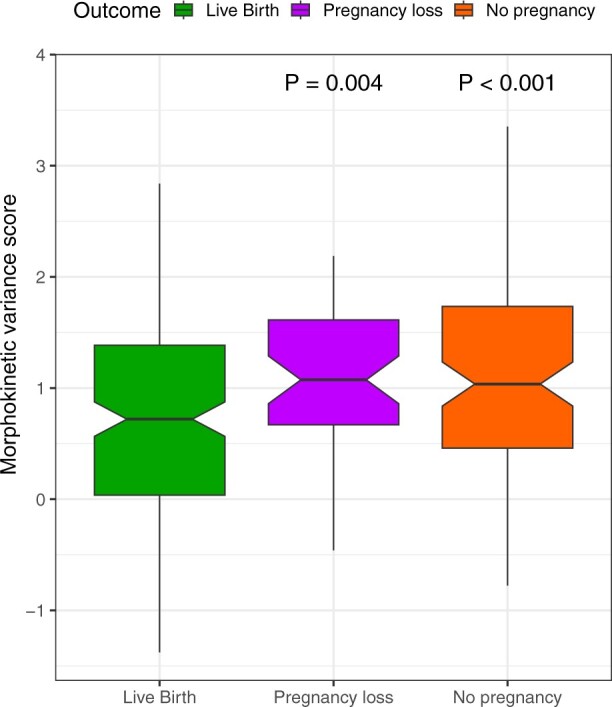
**Morphokinetic variance scores of euploid embryos**. Comparison of morphokinetic variance among euploid embryos that resulted in live birth, no pregnancy, or pregnancy loss. The live birth group had a lower morphokinetic variance score compared to the pregnancy loss and no pregnancy groups.

When analysing the MVS among the groups, euploid embryos leading to live birth had a significantly lower MVS compared to pregnancy loss and no pregnancy groups (*P* =0.004 and *P* < 0.001, respectively) (Fig. 2).

**Figure 3.**
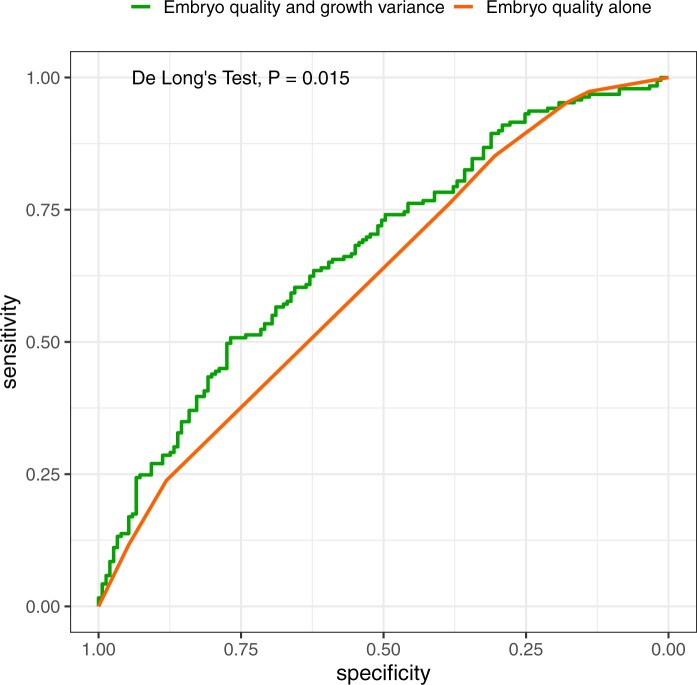
**Receiver operating characteristic curves of the explored models**. Receiver operating characteristics (ROC) of models using embryo quality (inner cell mass (ICM) and trophectoderm (TE) grading, red line) versus the model using embryo quality and morphokinetic variance (green line). The addition of morphokinetic variance significantly improved the area under the curve (AUC) of the model (0.67 vs 0.62, *P* = 0.015).

**Figure 4.**
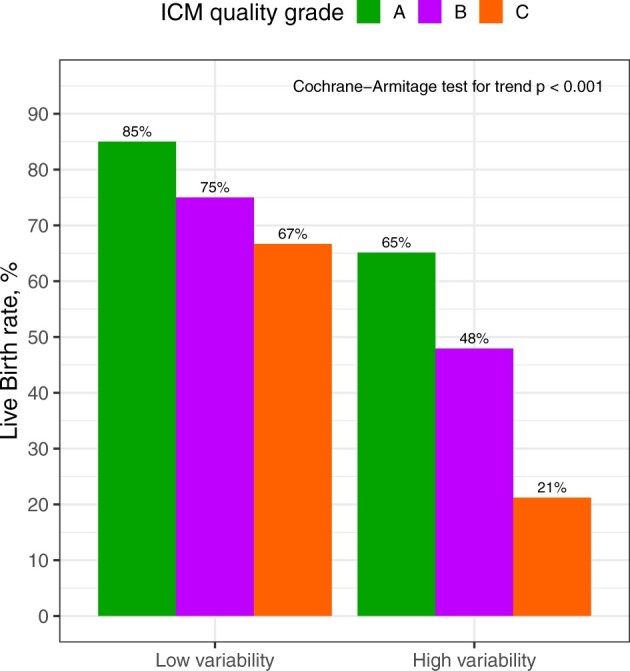
**Live birth rates stratified according to inner cell mass (ICM) quality grade and morphokinetic variance score**. The morphokinetic variance score cut-off of ≤0.3 was chosen as the point maximizing the negative predictive value in this dataset. Bars indicate the increased percentage in live birth rates for blastocysts in the groups of low compared to high morphokinetic variance scores.

The addition of the MVS to TE and ICM grades significantly improved the AUC of the model (AUC: 0.67 vs 0.62, *P* = 0.015, Fig. 3). Importantly, the addition of static morphokinetic time points with known correlations with implantation success (t8, tM, tB) did not significantly improve the baseline model including morphology parameters (AUC: 0.63 vs 0.62, *P* = 0.768). To account for over-optimism in estimated AUC values, repeated 5-fold cross-validation was applied. Optimism-adjusted AUC values showed that when morphokinetic variance was added, the model significantly outperformed the regular morphology base model (AUC: 0.64 ± 0.08 vs 0.60 ± 0.07, *P* < 0.001). A cut-off was determined for the MVS (0.3) that maximizes selection of embryos with low live birth potential to demonstrate the utility of MVS. When blastocysts were stratified by ICM grade and variance category (low vs high MVS, cut-off: 0.3), there was a significant trend towards lower live birth rates for embryos with higher variance (Cochrane-Armitage test for trend, <0.001, Fig. 4) compared to embryos with low variance. This trend was significant for all ICM grades but more prominent for blastocysts with poor-quality ICM. About 20% higher live birth rates were obtained for blastocysts of ICM type A in the group of low MVS compared to high MVS group (85% vs 65%, respectively). The absolute difference in live birth rates between low- and high-variance embryos was higher for blastocysts with poorer quality ICM (75% vs 48% and 67% vs 21% for ICM grades B and C, respectively) (Fig. 4, Supplementary Table S1). Post hoc calculations showed adequate power for both mixed-effects model (estimated power: >99%) and multivariable regression models (estimated power: 94%). An online calculator was built and deployed for research purposes and to allow the calculation of percentiles and assessing the MVS in other centres with ease: https://artfertilityclinics.shinyapps.io/Morphokinetics/.

## Discussion

In the present study including 340 euploid SET, TLM was utilized to analyse embryo morphokinetics to create a reference curve chart capable of identifying euploid embryos with the highest potential for live birth. Our results revealed that embryos with erratic morphokinetic patterns as measured with MVS were less likely to result in successful pregnancies. MVS was an independent marker of embryonic competence, additive to already established competence markers such as ICM and TE quality. The new metric showed that transfers of euploid blastocysts resulting in live births had a lower variance score than those resulting in pregnancy loss or no pregnancy.

Morphological grading is the most used method to evaluate the reproductive success of the embryos (Alpha Scientists in Reproductive Medicine and ESHRE Special Interest Group of Embryology *et al.*, 2011). However, embryo development is a dynamic process and significant events may be missed when relying solely on static observational time points (ESHRE Working Group on Time-Lapse Technology *et al.*, 2020). Some authors have reported the additional value of TLM for embryo selection and clinical outcomes (Gazzo *et al.*, 2019), while others could not find a significant additive value over morphological assessment for pregnancy and live birth rates (Pribenszky *et al.*, 2017; Armstrong *et al.*, 2019; Ahlström *et al.*, 2022; Bamford *et al.*, 2022; Meng *et al.*, 2022). Instead of comparing pre-selected morphokinetic parameters individually, as has been carried out by previous authors, our model considers all parameters provided by the TLM of a particular embryo as a single longitudinal trajectory.

The use of TLM helped to investigate the embryo developmental dynamics with the accurate timing of events, such as multinucleation. Although multinucleation *per se* is not a sufficient marker for abnormality (Hashimoto *et al.*, 2016), it might decrease the rate of implantation after transfer, presumably from chromosomal aberrations (Ergin *et al.*, 2014; Aguilar *et al.*, 2016; Desch *et al.*, 2017; Sayed *et al.*, 2022). Moreover, a recent meta-analysis (Bamford *et al.*, 2022) has shown that multinucleation persisting to the four-cell stage can have some prognostic potential for aneuploidy. The relevance of our finding rests on the fact that, in euploid blastocysts, the presence of nuclear errors such as multinucleation at four-cell stage decreased the chances of live birth by 60% in a regression analysis model.

The significant correlation between blastocyst quality, euploidy, and implantation potential is well described (Hernandez-Nieto *et al.*, 2019; Boynukalin *et al.*, 2020; Abdala *et al.*, 2022). However, identifying the best quality blastocysts for transfer after PGT-A presents certain challenges. By using Gardner’s classification, blastocysts can be categorized as excellent, good, and poor, and it was demonstrated that even blastocysts classified as poor morphological quality may in fact be euploid and implant (Morbeck, 2017). In our study, applying the MVS to TE and ICM grades significantly improved the AUC of the model to live birth from 0.62 to 0.67. Notably, the addition of static morphokinetic time points (t8, tM, tB) which are known to correlate with implantation success (Dal Canto *et al.*, 2012; Chamayou *et al.*, 2013; Kirkegaard *et al.*, 2013; Rienzi *et al.*, 2019) did not enhance the baseline model that included TE and ICM grading. This suggests that a longitudinal assessment of embryonic morphokinetics, rather than relying solely on static time points may more effectively identify embryos with the highest live birth potential. Moreover, the stratified model by ICM quality confirmed that embryos with a higher variance score had lower live birth rates. Interestingly, the lower the quality of the ICM, the higher the contribution of the MVS to live birth. For instance, good-quality blastocysts of low MVS category had 20% higher live birth rates (85%) compared to those of high variance score (65%). However, poor-quality blastocysts categorized as low MVS had 46% higher rates of live birth compared to those with low variance. This indicates that the impact of applying this variance score in euploid blastocysts is even more pronounced for the selection of poor-quality blastocysts. This could be because it is challenging for embryologists to ascertain the potential of implantation of a poor-quality blastocyst due to their depleted morphology. Even when euploid, poor-quality blastocysts generally have a lower implantation potential (Irani *et al.*, 2017; Suzuki *et al.*, 2024), likely due to factors unrelated to their nuclear constitution. Therefore, while the chance of selecting a euploid blastocyst that will implant from those graded ICM A/B is approximately one out of two, this likelihood decreases significantly with blastocysts of ICM grade C. Although several models have been developed to date (Kan-Tor *et al.*, 2020; Miyagi *et al.*, 2020; Duval *et al.*, 2023), none have focused on ranking poor embryos. Utilizing models such MVS would assist embryologists in aspects beyond their capabilities in identifying poor-quality blastocysts with the highest chances of success.

Several observational studies have elucidated that the pace of early embryonic divisions plays a key role on embryonic stability (Bartolacci *et al.*, 2021). Slow embryo development at cleavage stages can result in delayed blastulation associated with poor reproductive outcomes (Petersen *et al.*, 2016; Zaninovic *et al.*, 2017; Fishel *et al.*, 2018; Wu *et al.*, 2020; Abdala *et al.*, 2022). Prolonged cell cycles in the first stage of human embryo development are hypothesized to be associated with DNA repair mechanisms, erroneous attachment of chromosomes to the spindle apparatus, or inadequate completion of preceding cell cycle phases. On the contrary, a short cell cycle may be insufficient to allow complete DNA replication and repairs prior to chromosomal alignment and is rather related to inadequate cell cycle checkpoints (Ramos and De Boer, 2011; Coticchio *et al.*, 2021). When the checkpoint mechanisms detect deficiency with the DNA, the cell attempts to either complete DNA replication or repair the damaged DNA, slowing down cell division. If the damage is irreparable, the cell may undergo apoptosis and be expelled as fragmentation (Cecchele *et al.*, 2022). If the embryo contains partial amounts of aneuploid cells (mosaic), these cells might be expelled to rescue the embryo from aneuploidy. These events probably cause delayed blastulation, since it decreases the total contributing cells to develop the blastocyst (Lagalla *et al.*, 2017; Yang *et al.*, 2021; Currie *et al.*, 2022).

Since our analysis included only euploid embryos, those presenting high-MVS (abnormal kinetic pace), could have been submitted to mechanisms of correction during embryonic divisions to sustain euploidy at the blastocyst stage. Their association with pregnancy loss leads to the supposition that there are underlying mechanisms having an impact on genomic instability at later stages of blastocyst development, post-embryo transfer. Failed implantation or pregnancy loss of euploid embryos could be due to factors independent of the original embryonic chromosomal constitution, such as embryo metabolism and/or mitochondrial dysfunction, or other cytoplasmic-related elements. Several studies have found a correlation between embryo viability and metabolites absorbed or secreted by the embryo (Inoue *et al.*, 2021; Venturas *et al.*, 2023). It remains to be clarified if altered embryonic metabolism could be related with implantation failure or embryo demise post-implantation. Another factor leading to pregnancy loss could be related to telomere length instability. Telomeres are transcriptionally inactive genomic areas, which, if shortened, are associated with chromosomal rearrangements and genomic instability. Studies have already shown an association of spontaneously lost pregnancies with shortened telomeres in euploid embryos (Huleyuk *et al.*, 2018). The pace of embryonic division could thus be also influenced by irregular or faulty telomeres. However, while the role of telomeres in postnatal life has been extensively studied, the role of telomere length in prenatal development is still poorly understood (Orvieto *et al.*, 2020).

The capability of prioritizing embryos for transfer based on assessments of their competence is of ultimate importance to reduce the time to pregnancy. Our MVS showed good potential to be of additive value to the morphological grading and may assist on the task of prioritization. The strong points of our research were the inclusion of euploid embryos, reporting live birth rates and most of the significant morphological and morphokinetic variables being used for embryo assessment. A reason for caution for the interpretation of our results is the exclusion of IVF cases, which limits for now the utility of the model to only ICSI-derived embryos. Further studies should be carried out to reproduce the utility of these reference ranges and the association of MVS with various clinical outcomes. Ideally, future studies would require a prospective concordance analysis where the first euploid blastocyst to transfer is chosen based on the highest rank given by the MVS model.

## Conclusion

We propose a new metric utilizing morphokinetic assessment for selection of embryos for transfer which is associated with embryonic potential beyond implantation. The MVS seems to have additive potential to the established embryo selection parameters and should be studied further in external cohorts.

## Supplementary Material

hoae059_Supplementary_Data

## Data Availability

The data underlying this article will be shared on reasonable request to the corresponding author.
