# Supplementary material for: Steady morphokinetic progression is an independent predictor of live birth: a descriptive reference for euploid embryos
Source: Hum Reprod Open. 2024 Oct 10;2024(4):hoae059. doi: 10.1093/hropen/hoae059 (PMC11540439; doi:10.1093/hropen/hoae059)
Supplement: hoae059_Supplementary_Data [file hoae059_supplementary_data.zip › HRO-24-0129-R2-SuppTable1.docx]

**Supplementary Table S1. Live birth rates according to inner-cell mass quality grade and variance score group.**

| ICM quality grade | Variance group |  | Live Birth | Total | Rate |
| --- | --- | --- | --- | --- | --- |
| A | Low variance |  | 17 | 20 | 0.85 |
| B | Low variance |  | 54 | 71 | 0.76 |
| C | Low variance |  | 2 | 3 | 0.67 |
| A | High variance |  | 28 | 43 | 0.65 |
| B | High variance |  | 81 | 170 | 0.48 |
| C | High variance |  | 7 | 33 | 0.21 |
