# Supplementary material for: Steady morphokinetic progression is an independent predictor of live birth: a descriptive reference for euploid embryos
Source: Hum Reprod Open. 2024 Oct 10;2024(4):hoae059. doi: 10.1093/hropen/hoae059 (PMC11540439; doi:10.1093/hropen/hoae059)
Supplement: hoae059_Supplementary_Data [file hoae059_supplementary_data.zip › Supplementary File S1.docx]

**Supplementary File-Figures**

**Explanation of morphokinetic variance concept and use of online calculator**

Growth reference charts are commonly used in clinical practice. Some examples include the monitoring of fetal growth using growth reference charts or the postnatal growth of newborns. These reference charts are extremely useful for assessing the normality of growth. In the case of embryo growth, our modeling did not involve a biometric measurement as in the previous examples, but rather the time it took the embryo to reach certain morphokinetic time points. After creating reference charts and plotting individual morphokinetic trajectories, visual inspection suggested that embryos with erratic morphokinetic patterns of development- i.e., those that were delayed at certain stages and then caught up more quickly at other stages - were more likely to fail than those with steady (monotonous) morphokinetic progression. Based on this observation, we wanted to summarize this pattern with a new metric.

Variance in essence reflects the steadiness of embryos morphokinetic progression. Variance is a measure of how much a set of values scatters around their mean (Figure S1).


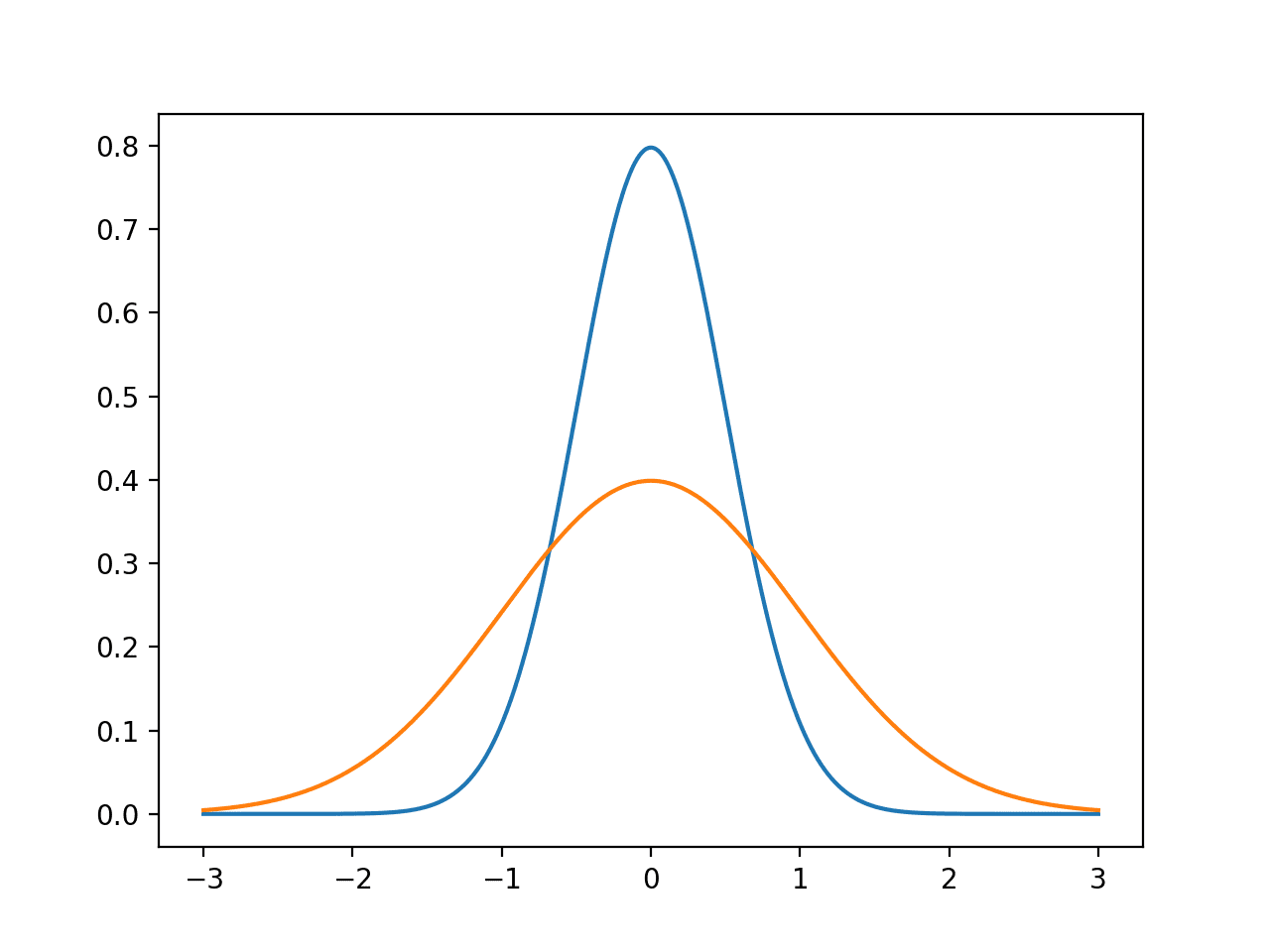


**Figure S1.** Density plots for two sets of values showing lower (blue) and higher variance (orange). The set with lower variance scatter less around their mean and higher variance set scatter a lot more.

In this setting an embryo with a high variance will show significant changes of its developmental milestone Z-scores or percentiles. Embryos that will reach some developmental milestones too quickly and then showing delayed, showing uneven progression may classify as high-morphokinetic variance. Not unlike untrained marathon runners who have occasional sprints and fast movement and then significant delays to catch their breaths and rest.


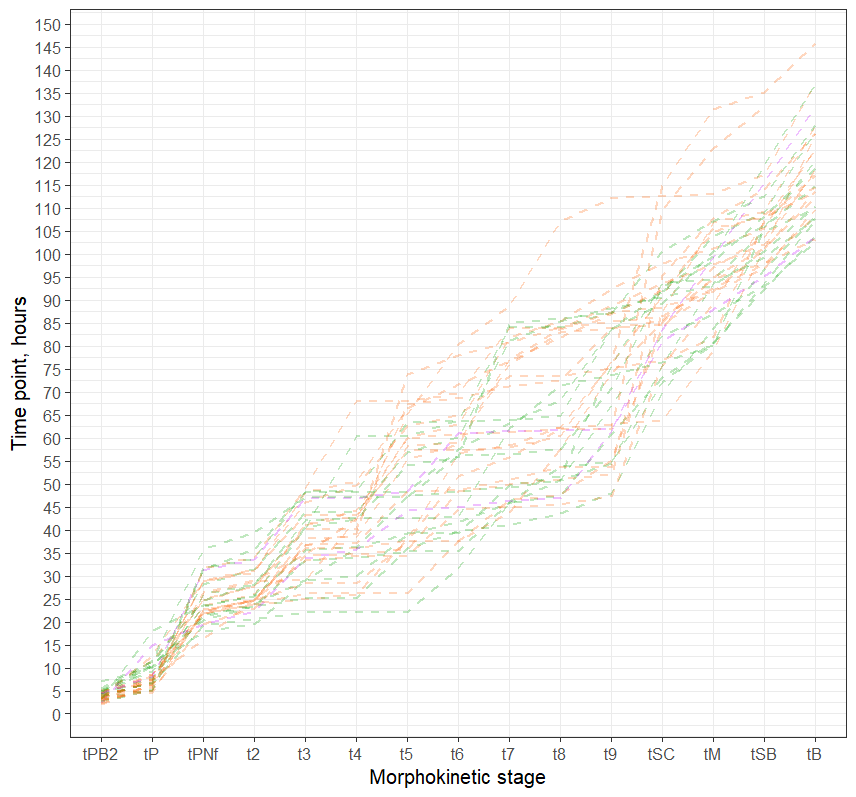


**Figure S2.** Longitudinal trajectory for morphokinetic milestones of a group of embryos with high-variance. Green, purple and orange dashed line show embryos that ended in live birth, pregnancy loss and no pregnancy, respectively.

An embryo with low-variance on the other hand will show a steadier morphokinetic progression and less scattering around its mean Z-score or percentile. Using the same analogy, it will be like a trained marathon runner who has a steady pace whether it be slow or fast. Figure S1 and S2 show a set of embryos with high and low morphokinetic variance. Note how tightly packed the longitudinal growth is in low-variance embryos compared to high-variance ones which have significant scattering around their mean trajectory.


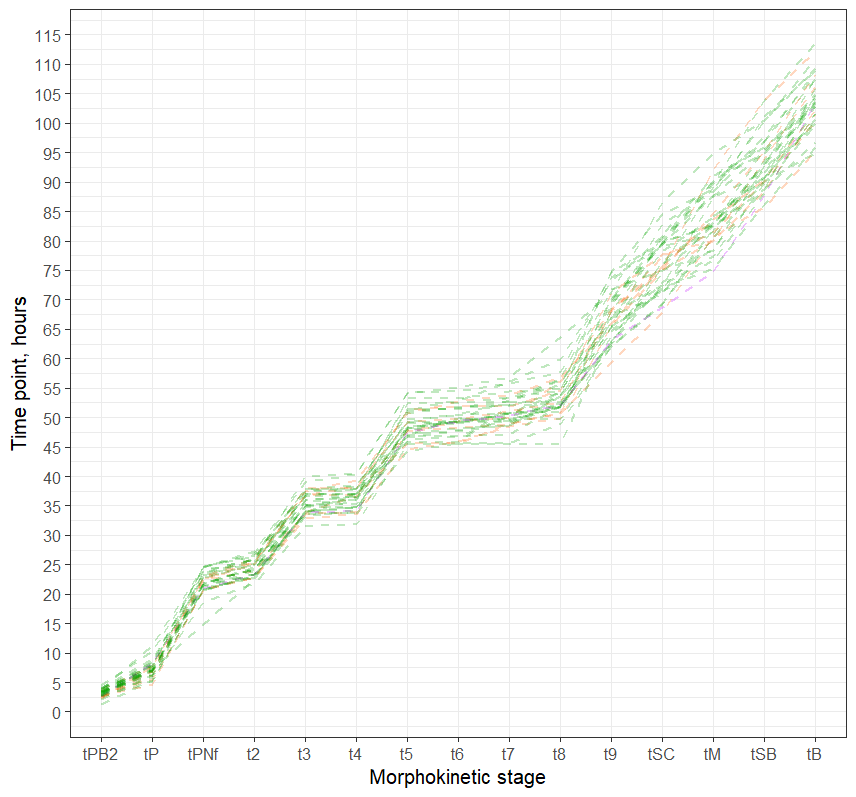

**Figure S3.** Longitudinal trajectory for morphokinetic milestones of a group of embryos with low-variance. Green, purple and orange dashed line show embryos that ended in live birth, pregnancy loss and no pregnancy, respectively.

The following Figure S4 show the calculation of morphokinetic variance for two examples of one low (Figure S4a) and one high (Figure S4b) variance embryo. The calculator shows the percentiles of each morphokinetic milestone along with calculated morphokinetic variance score (MVS). (<https://artfertilityclinics.shinyapps.io/Morphokinetics/>).

**Figure S4.** **Morphokinetics calculator ART Fertility clinics:**


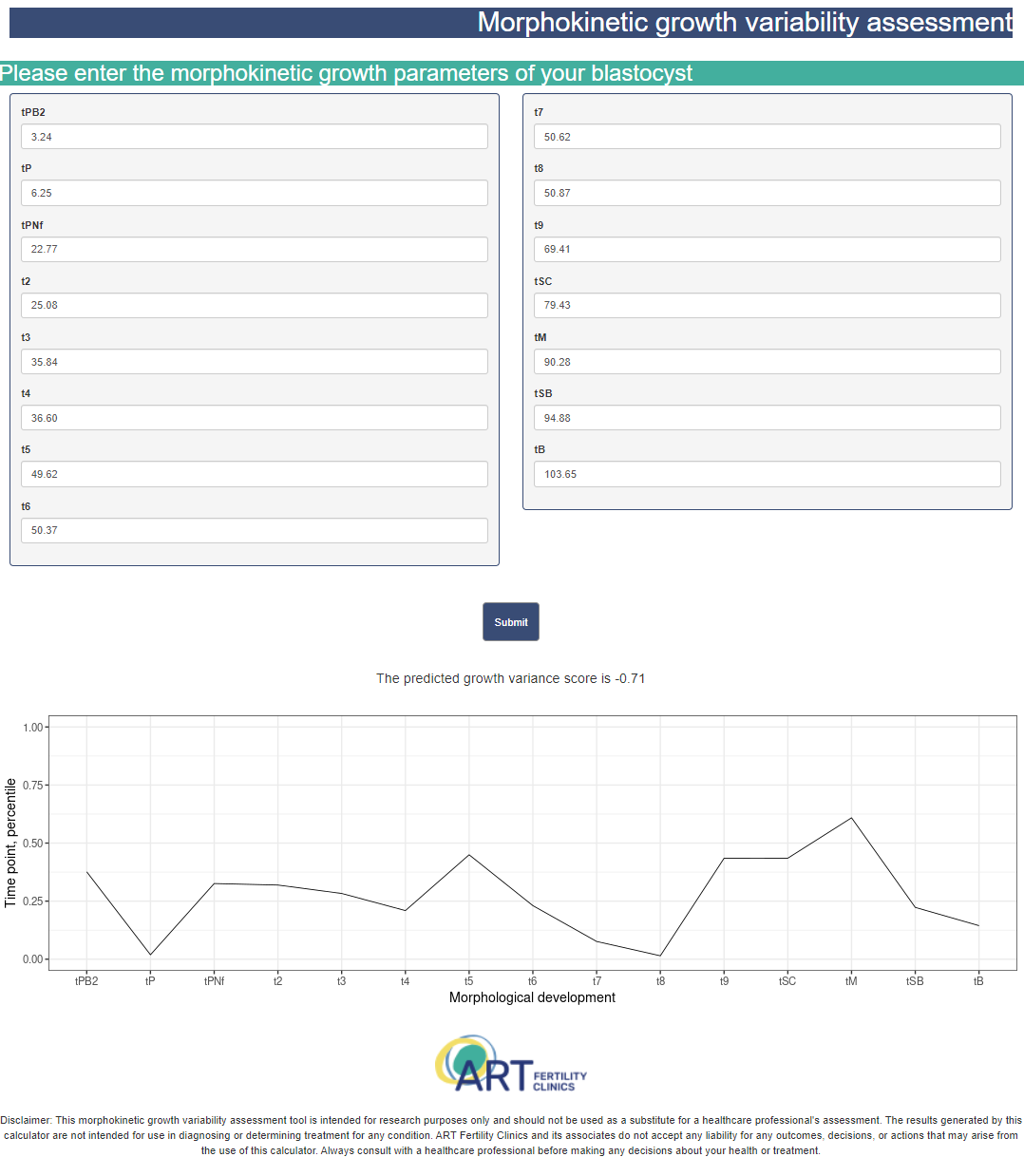


**Figure S4a.** Screenshot of calculator webpage in which a low-variance embryo percentiles are calculated. Graph shows the percentile of each time point and text above gives the calculated variance score.


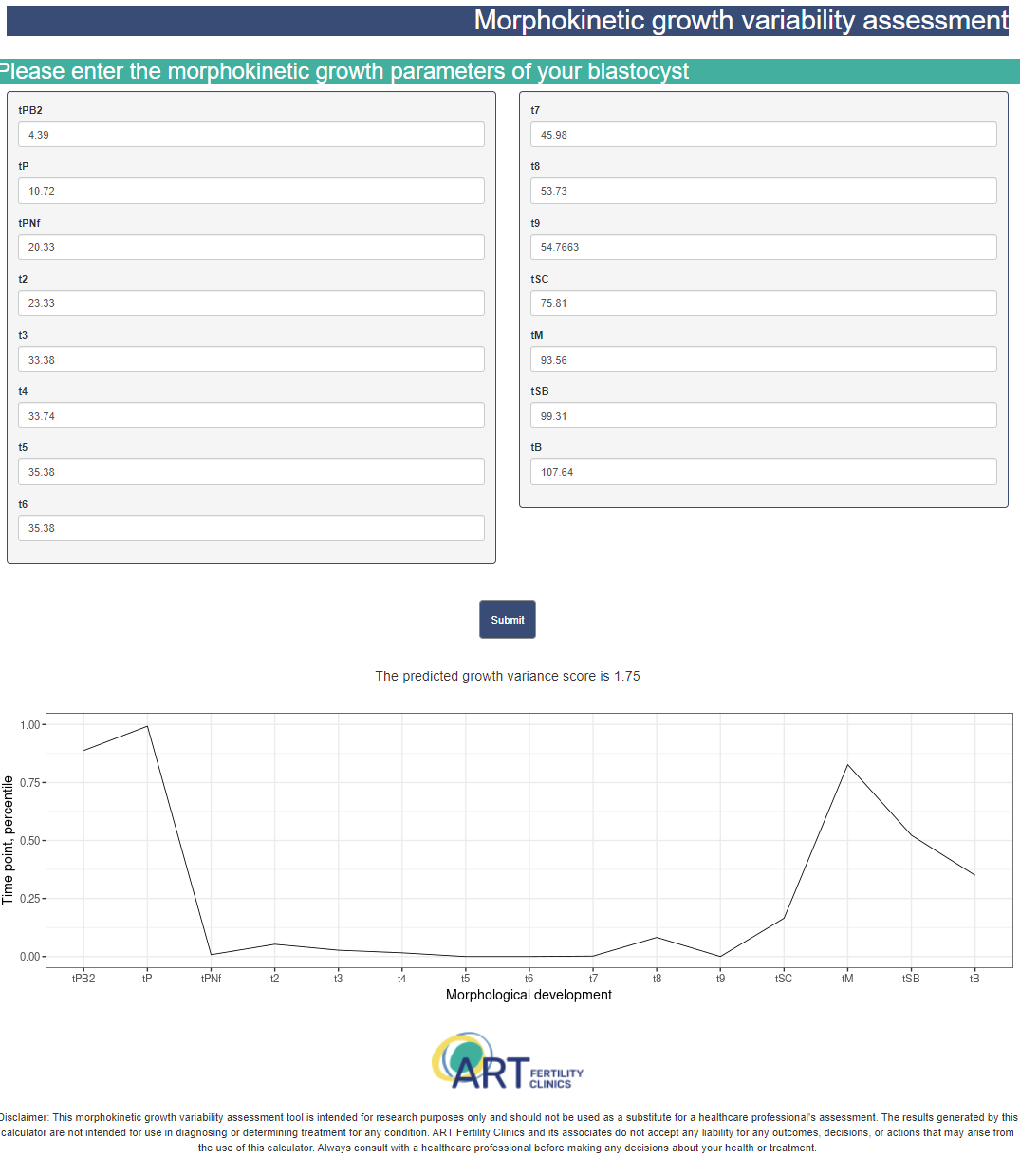


**Figure S4b.** Screenshot of calculator webpage in which a high-variance embryo percentiles are calculated. Graph shows the percentile of each time point and text above gives the calculated variance score.
